# Supplementary material for: The dark exciton ground state promotes photon-pair emission in individual perovskite nanocrystals
Source: Nat Commun. 2020 Nov 26;11:6001. doi: 10.1038/s41467-020-19740-7 (PMC7691346; doi:10.1038/s41467-020-19740-7)
Supplement: Supplementary file 1 — Supplementary Information [file 41467_2020_19740_MOESM1_ESM.pdf]

Supplementary Information for

The dark exciton ground state promotes photon-pair emission in individual perovskite nanocrystals

Philippe Tamarat, Lei Hou, Jean-Baptiste Trebbia, Abhishek Swarnkar, Louis Biadala, Yann Louyer, Maryna I. Bodnarchuk, Maksym V. Kovalenko, Jacky Even, Brahim Lounis

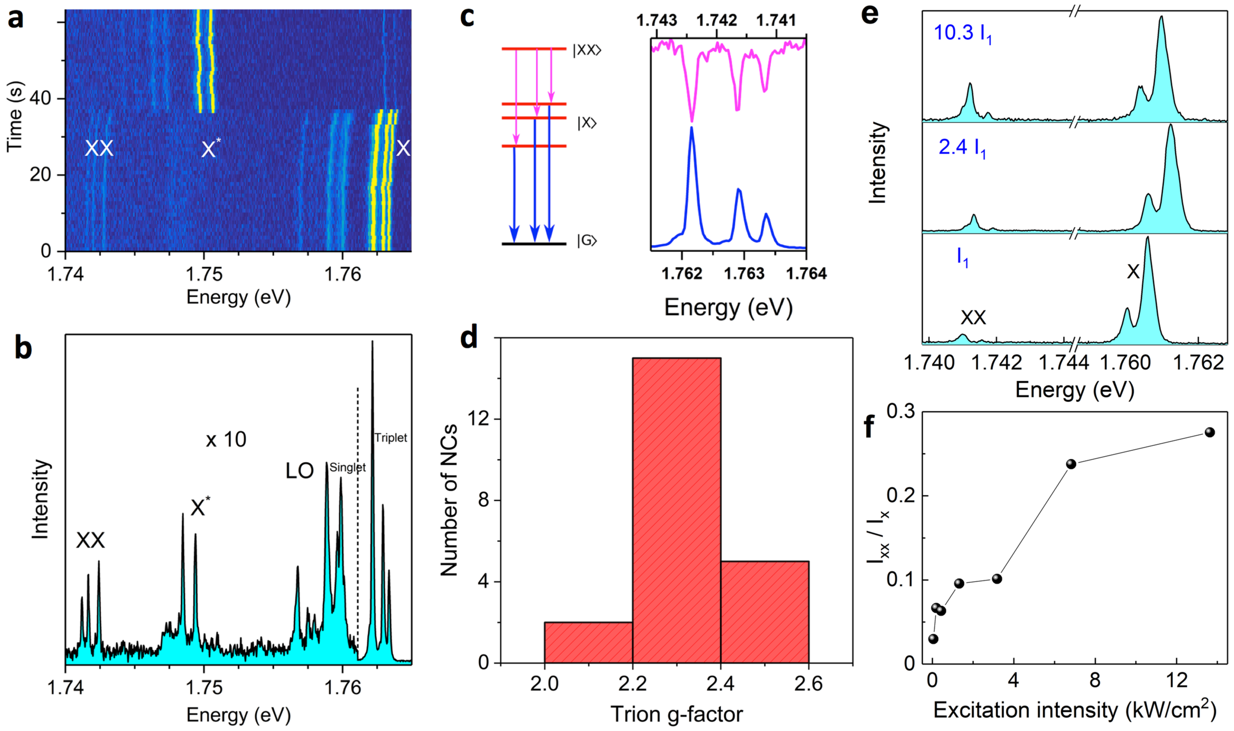

Supplementary Figure 1: Spectral signatures of biexciton and trion in single $CsPbI_3$ NCs. **a**, Spectral trajectory of a single NC at 4 K and under a magnetic field of 7 T, built with 63 consecutive PL spectra, each recorded over 1 s at an excitation intensity of ~50 W cm^{-2}. After 38 s, it shows a switch between exciton (X) emission, together with biexciton (XX) emission, and trion (X*) emission. **b**, PL spectrum of the same single NC, built from the trajectory **a** by shifting the spectra in order to eliminate the ZPL spectral diffusion. It displays the spectral fingerprints of the exciton band-edge exciton fine structure, together with LO-phonon replica (LO), biexciton and trion emissions. The correspondence between the biexciton spectral structure and the exciton multiplet is evidenced in **c**. As for all studied NCs, the trion structure presents two characteristic Zeeman components under magnetic fields. **d**, The narrow distribution of the trion Landé factor centered at ~2.3 is very similar to the one obtained with single $CsPbBr_3$ [1] and $FAPbBr_3$ [2] NCs and suggests that the electron g-factor is close to 2 (that is, the value for a free electron), while the hole g-factor is much smaller ($\lesssim 0.3$). **e**, PL spectra of a single NC at three different excitation intensities, with I_1 = 1.3 kW cm^{-2}. **f**, The ratio of integrated intensities of the XX and X spectral structures of this NC grows as a function of the excitation intensity, which points to a super-linear dependence of the XX structure upon excitation intensity.

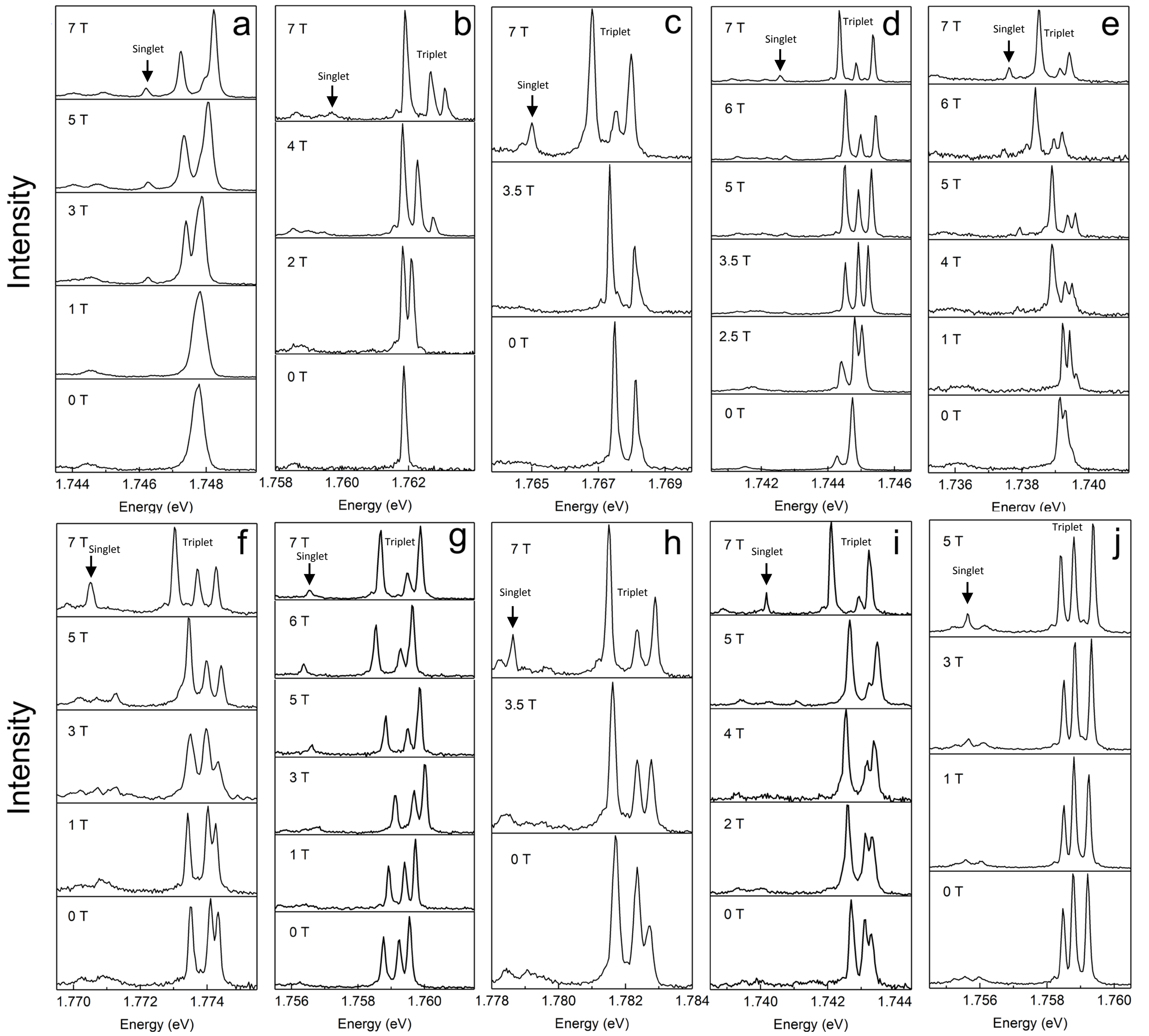

Supplementary Figure 2: Evolution of the PL spectrum of ten single $CsPbI_3$ NCs with magnetic field.

The PL spectra are recorded at 4 K at an excitation intensity of ~50 W cm^{-2}. When increasing the magnetic field, a new emission line emerges several meV below the bright triplet, as a signature of magnetic brightening of a low-lying dark exciton state in these perovskites, attributed to the singlet state. The magnetic field also splits to bright triplet structure, so that the spectra at 7 T display the entire band-edge exciton fine structure. **a**, **b**, PL spectra displaying one ZPL in zero field. **c**, **d**, PL spectra displaying two ZPLs in zero field. **e**, **f**, **g**, **h**, **i**, **j**, PL spectra displaying three ZPLs in zero field. The frames **a**, **c**, **f** correspond to the NCs whose PL spectra at 0 T and 7 T are displayed in Fig. 2.

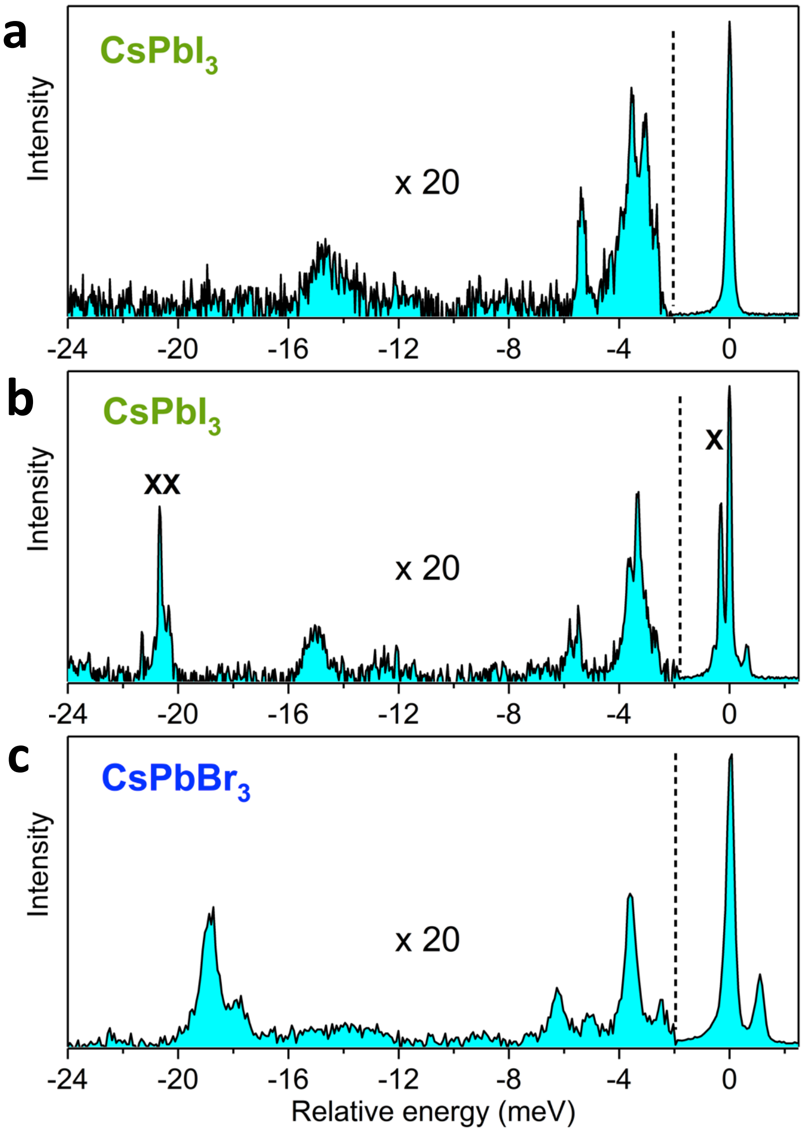

Supplementary Figure 3: LO-phonon spectrum of $CsPbI_3$ NCs and $CsPbBr_3$ NCs.

High quality PL spectra of single NCs at 4K reveal a large set of LO phonon replica of the ZPL multiplets. The excitation intensity is ~100 W cm^{-2}. **a**, **b**, PL spectra of two different $CsPbI_3$ NCs: one with a single ZPL (**a**), one with exciton and biexciton ZPL triplets (**b**). The structure of the LO phonon replica comprises three LO phonon peaks with red shifts in the range 3.0 - 5.4 meV with respect to the ZPL, followed by three larger sidebands, whose dominant component is red-shifted by ~15 meV. There are thus many combinations for the occurrence of two LO modes whose energy difference matches the bright-dark splitting. Moreover, all phonon modes do not appear in the PL spectrum due to specific selection rules of photoluminescence. A thorough determination of the phonon modes requires combining these results with those deduced from other techniques such as infrared absorption spectroscopy[3], Raman spectroscopy and inelastic neutron scattering [4]. The large sidebands can be assigned to bundles of finely separated low-energy lattice modes corresponding to stretching and bending vibrations of the PbI_3 network coupled to the motion of the cation. It is worth noting that this set of phonon replica is very similar to that of $CsPbBr_3$ NCs (**c**) within a scale factor ~1.25 that matches the ratio of square roots of atomics masses of I and Br atoms. This is consistent with LO phonon modes associated to vibrations of the lead halide network. Yet, significant differences show up with the LO-phonon spectrum of $FAPbI_3$ NCs, which presents three bands at ~3.5 meV, 11 meV and 15 meV [5]. This indicates that the vibrations of the PbI_3 network are strongly influenced by the rigid-body motion of a large cation such as formamidinium [3].

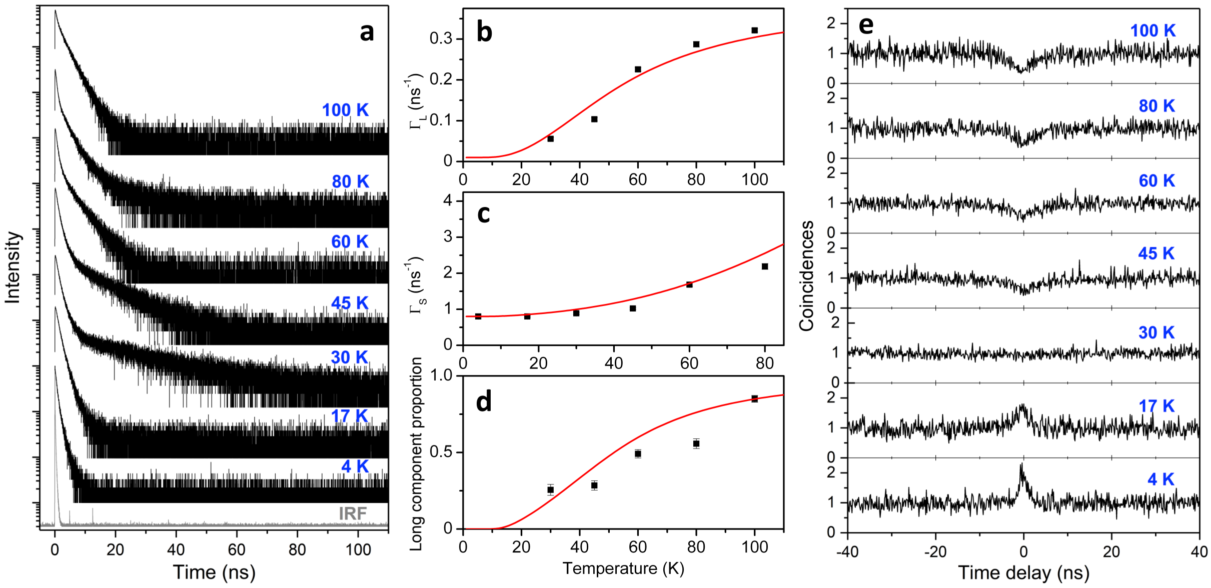

Supplementary Figure 4: Thermal mixing of dark and bright states.

a, Evolution of the PL decay of a $CsPbI_3$ NC with temperature. The average number of exciton per pulse is $\langle N \rangle$=0.2. The instrument response function (IRF) is shown in grey. Temperature dependence of the long component decay rate Γ_L (**b**), the short component decay rate Γ_S (**c**) and the proportion of photons emitted in the long decay component (**d**). The red curves are simulations with a two-phonon thermal mixing model [2,5] with transition rates between bright and dark sublevels $\gamma_\uparrow = \gamma_0 N_2(N_1+1)$ and $\gamma_\downarrow = \gamma_0 N_1(N_2+1)$, where $N_i = 1/[\exp(E_i/k_B T) - 1]$ are the Bose-Einstein phonon numbers (i = 1, 2), and γ_0 is a characteristic two-phonon mixing rate, taking Γ_B= 0.8 ns^{-1} and Γ_D= 0.01 ns^{-1} for the bright and dark exciton recombination rates, respectively, γ_0= 1 ns^{-1}, E_1= 3 meV, E_2= 5.4 meV. More refined models should take into account the whole distribution of phonon modes and the entire exciton fine structure in the two-phonon process. Furthermore, higher-order phonons may also contribute to the thermal mixing of bright and dark states. **e**, Histograms of time delays between consecutive photon pairs from the same $CsPbI_3$ NC, at various temperatures. Their evolution from bunching to antibunching with temperature is set by thermal mixing between the long-lived ground state and the bright states, which manifests in the evolution of the PL decay with temperature (**a**).

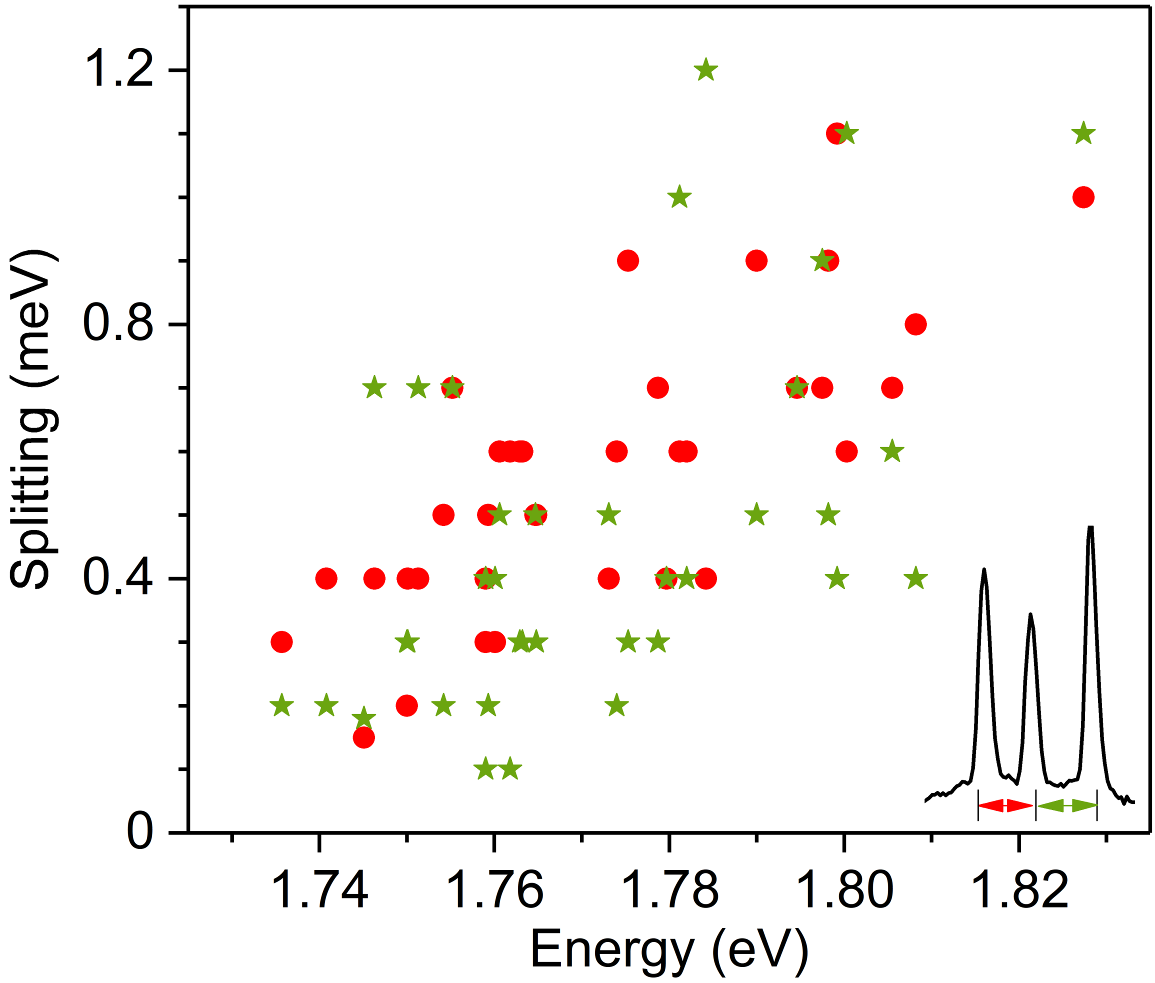

Supplementary Figure 5: Splittings within the bright triplet structure of $CsPbI_3$ NCs.

The splittings within the bright triplet structure are measured for 30 NCs displaying three-line PL spectra in zero field. The red (resp. green) points are the splittings between the two lowest (resp. highest) energy ZPLs. These splittings are plotted as a function of the exciton recombination energy, taken at the central ZPL.

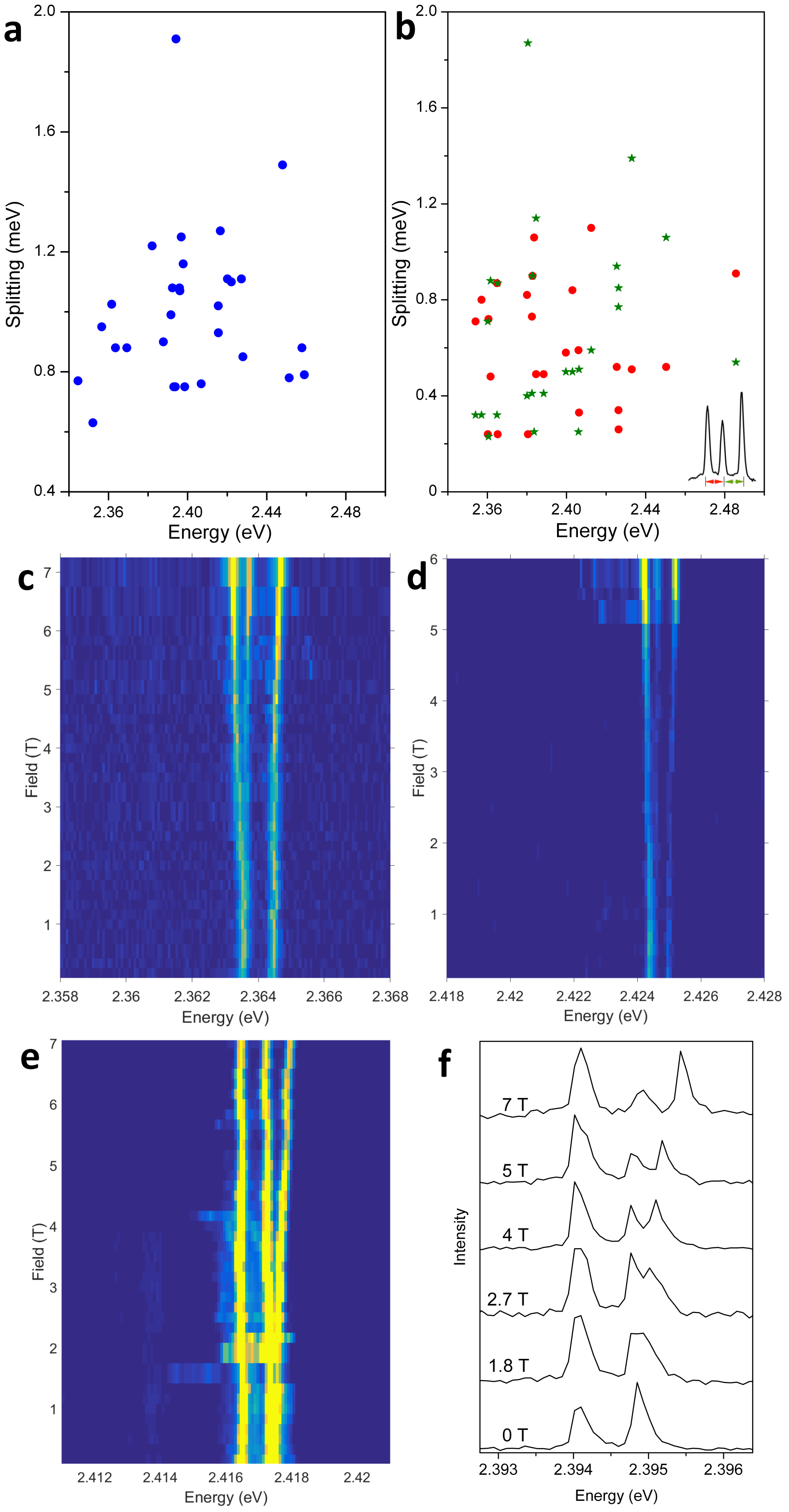

Supplementary Figure 6: Bright triplets splittings of $CsPbBr_3$ NCs.

a,**b**, Zero-field splittings within the bright triplets of single $CsPbBr_3$ NCs (with a size in the range 8-10 nm) as a function of exciton recombination energy, for the two-line PL spectra (a) and for the three-line PL spectra (b). **c**,**d**,**e**,**f** Magnetic field dependence of the bright triplet splittings of four single $CsPbBr_3$ NCs. The spectral trajectories (c) and (d) are those of two NCs for which the bright Z state is above the Zeeman-split X,Y states, while (f) and (g) show the PL spectra of two NCs for which the bright Z state is below the Zeeman-split X,Y states.

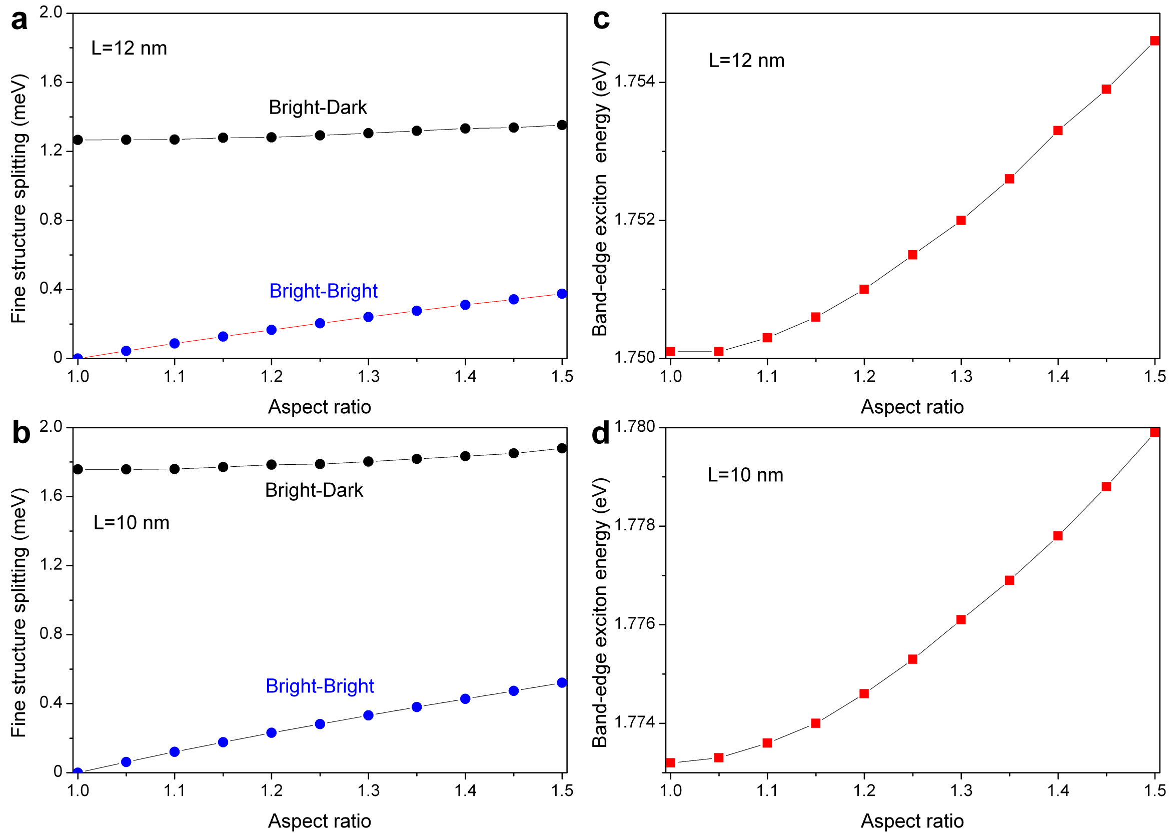

Supplementary Figure 7: Band-edge exciton energy and contribution of the long-range exchange interaction to the fine structure splittings in $CsPbI_3$ NCs.
a, **b**, Calculated fine structure splittings for tetragonal-shaped NCs as a function of the NC aspect ratio, for two different average NC lengths *L*. Bright triplet splittings (blue circles), bright-dark splittings (black circles). The contribution of dielectric confinement (see Supplementary Note 1) is not taken into account. **c**, **d** Calculated dependence of the exciton recombination energy on the aspect ratio. The effects of the aspect ratio on the emission energy remain limited to less than 3 meV for an aspect ratio less than 1.3, which corresponds to the major part (~90%) of the NCs according to their aspect-ratio distribution.

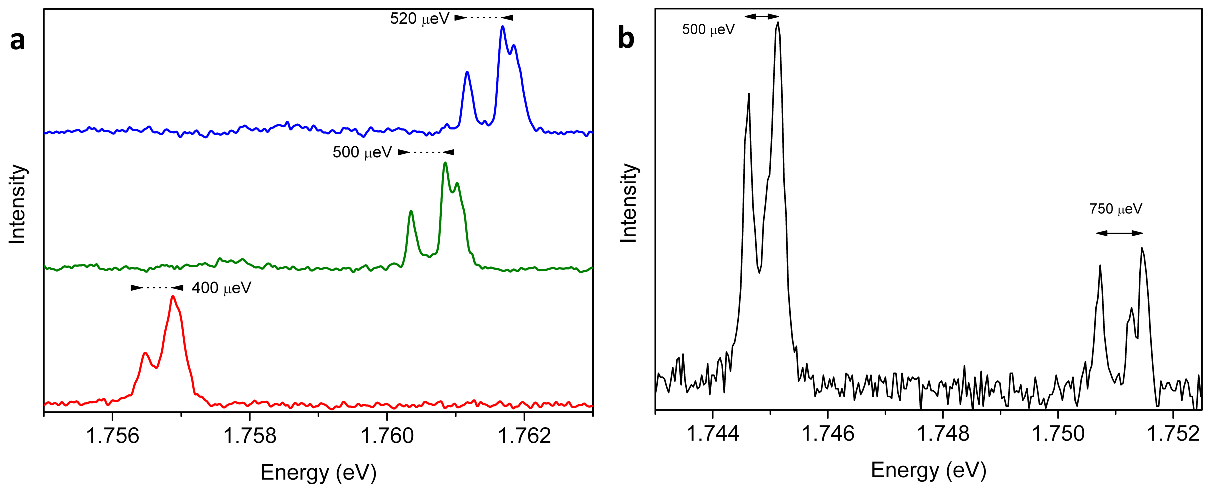

Supplementary Figure 8: Variation of the fine structure splitting after a spectral jump.

a, Selected PL spectra of a NC along its spectral trajectory, at 4K. The acquisition time of each spectrum is 1 s. Intensity offsets have been added for clarity. **b**, PL spectrum of another single NC, recorded over a period of 10 s during which a spectral jump occurred. We find that the fine structure splitting increases when the exciton recombination energy is higher, as observed in the case of $FAPbBr_3$ NCs [2].

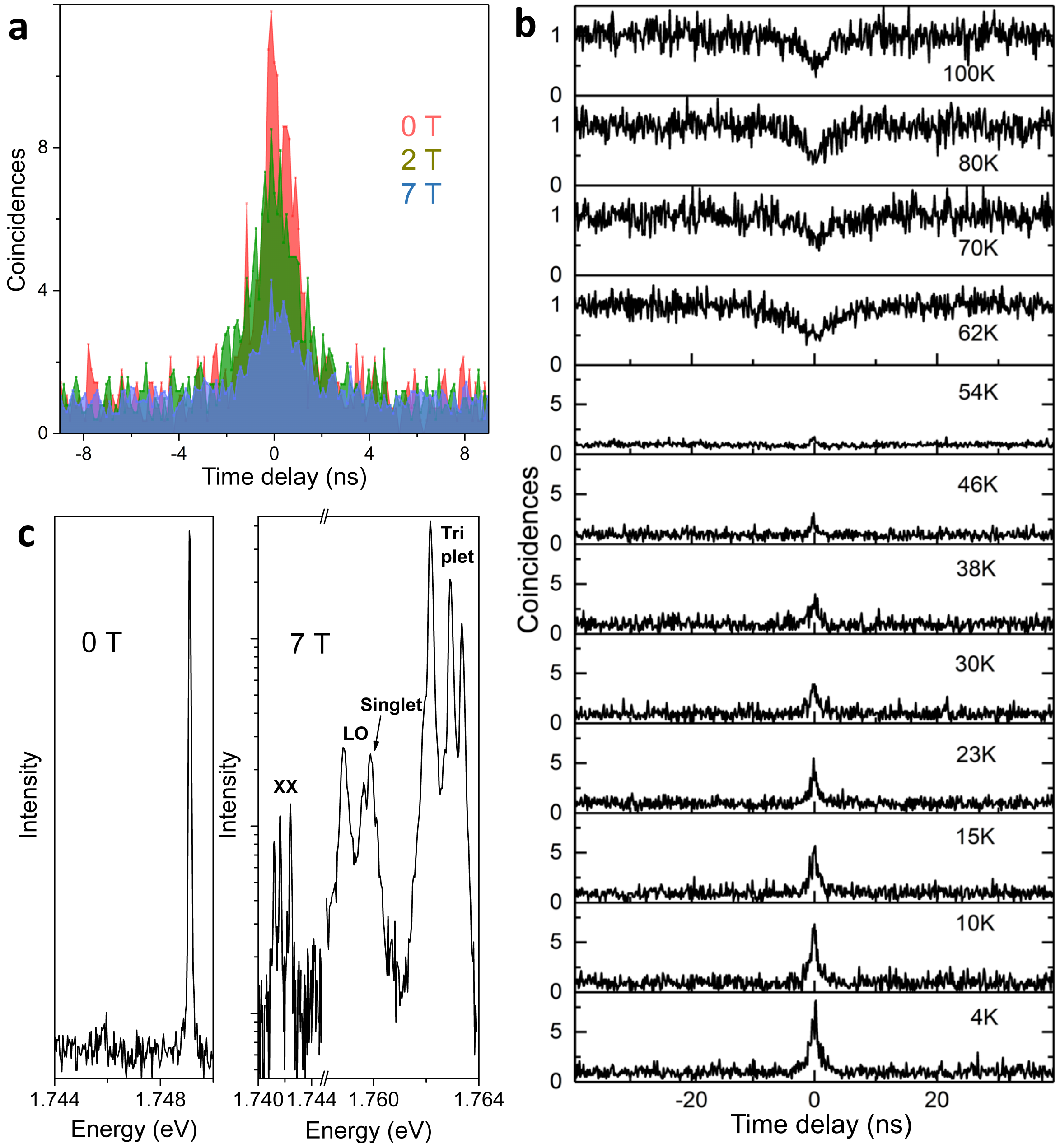

Supplementary Figure 9: Tuning the PL autocorrelation function of a $CsPbI_3$ NC with magnetic field and temperature.

The exciton shelving role played by the long-lived ground exciton state in the biexciton formation is altered by field-induced coupling or thermal mixing of bright and dark states, leading to a drop in photon bunching with increasing fields (**a**) or temperature (**b**). (**c**), PL spectrum of this NC at 0 T (linear scale) and at 7 T (logarithmic scale), where the singlet state emerges from the lowest-energy LO phonon replica. The biexciton structure is labeled XX. The excitation intensity is ~100 W cm^{-2}.

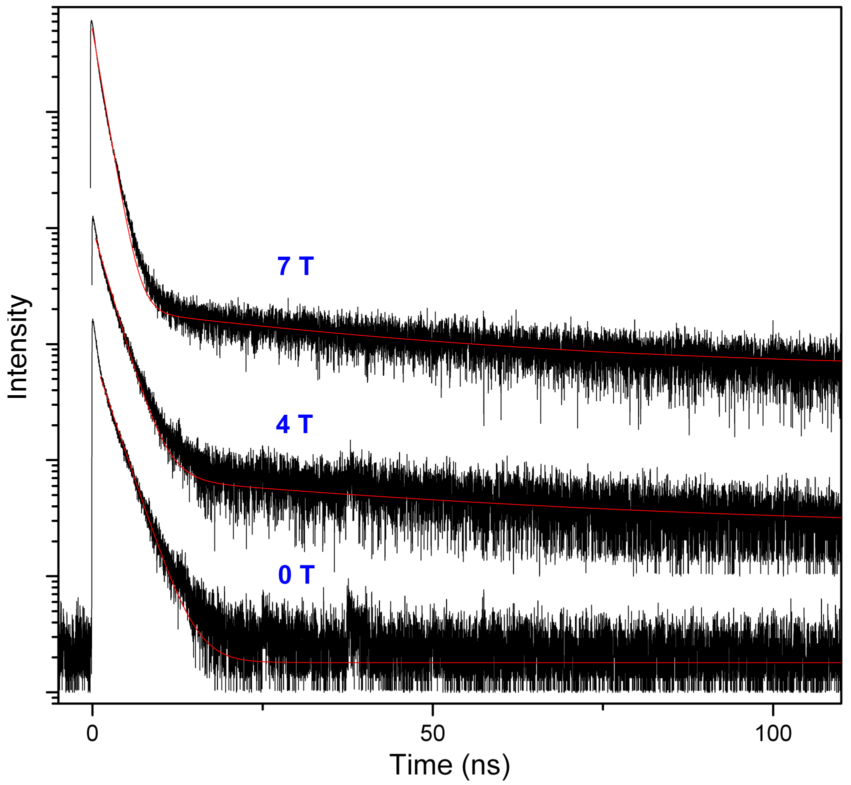

Supplementary Figure 10: Signature of the dark–bright magnetic coupling in the PL decay of a single NC.

PL decay of a single $CsPbI_3$ NC for various amplitudes of magnetic field, at a temperature of 4 K. The average number of exciton per pulse is $\langle N \rangle = 0.2$. In zero field, the PL decay has a lifetime of ~1 ns. Under magnetic field, the decay becomes biexponential. Its long time component shortens (lifetime ~50 ns at 4 T, ~38 ns at 7 T, deduced from the biexponential fits in red) and gains weight with increasing fields. Indeed, the magnetic coupling opens a radiative recombination channel for the ground exciton via admixture in the dark state of one of the bright triplet states.

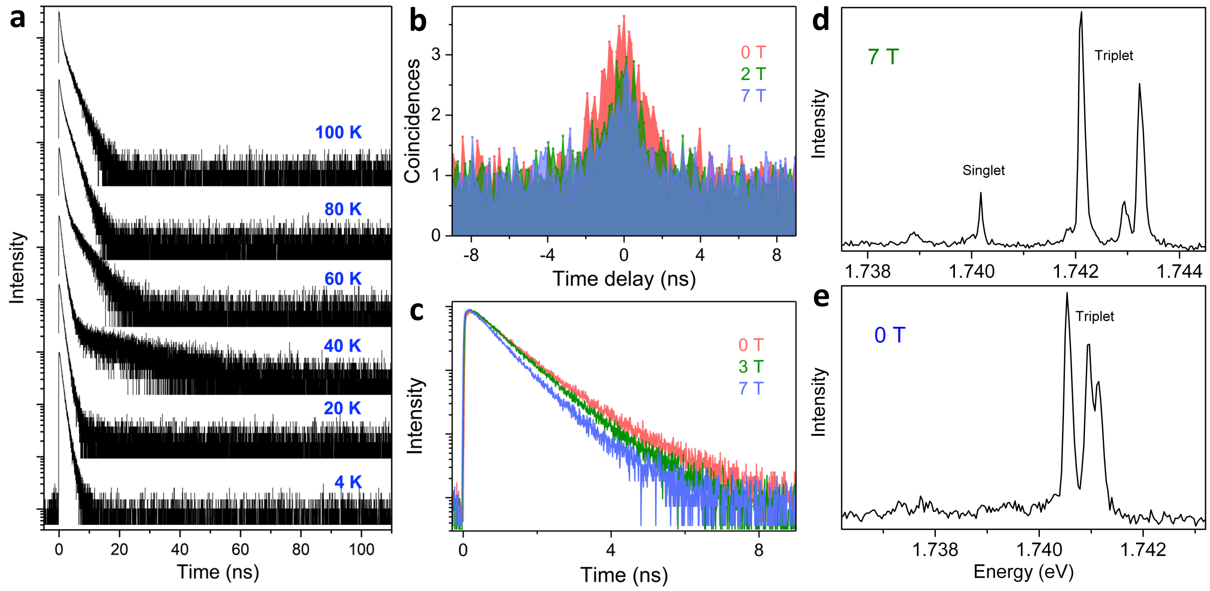

Supplementary Figure 11: Signatures of bright-dark thermal mixing and magnetic coupling for the NC of Fig. 5a.

a, Evolution of the PL decay with temperature. The average number of exciton per pulse is $\langle N \rangle$=0.2. **b**, Normalized photon coincidence histograms for various magnetic fields, at 4 K. The field-induced coupling between dark and bright states is also manifested in the evolution of the PL decay with magnetic field (**c**). Magnetic brightening of the dark exciton state for this NC is evidenced from the comparison of its PL spectra at 7 T (**d**) and at 0 T (**e**).

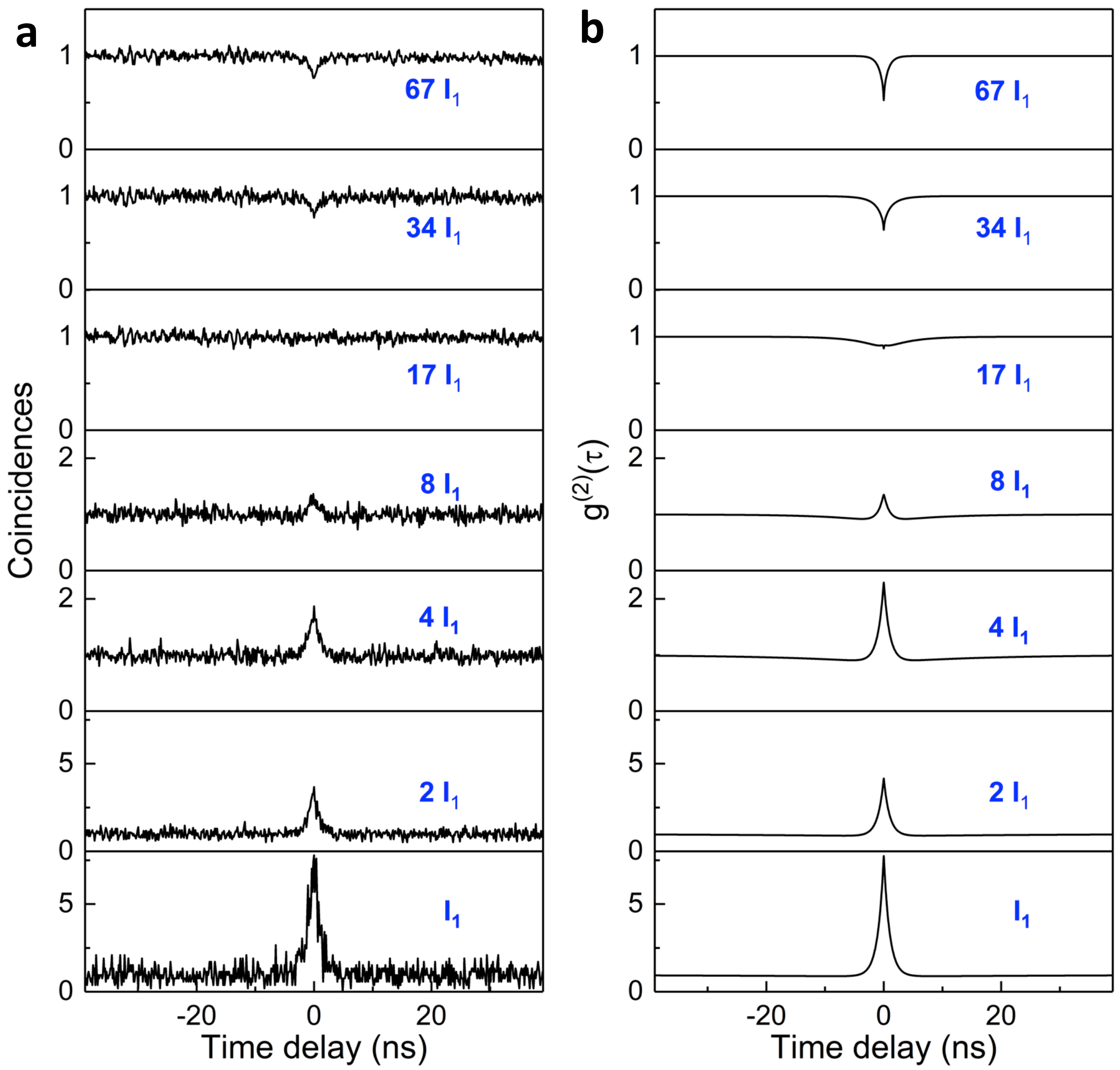

Supplementary Figure 12: Evolution of the autocorrelation function with increasing excitation intensities.

a, For a single $CsPbI_3$ NC at 4 K, evolution from bunching to antibunching as the excitation intensity increases, starting from I_1 ~100 W cm^{-2}. The coincidence histograms are well reproduced with numerical simulations (**b**) of the autocorrelation function (see Supplementary Note 2), taking $\Gamma_B = 1$ ns^{-1}, $\Gamma_D = 0.001$ ns^{-1}, $\gamma_0 = 0.1$ ns^{-1}, $\alpha = 3/4$ since the exciton fine structure is composed of three bright sublevels and one dark sublevel, $W = 0.012$ ns^{-1} for the lowest excitation intensity, $\eta_{XX} = 0.35$, $E_1 = 3$ meV, E_2 =5.6 meV.

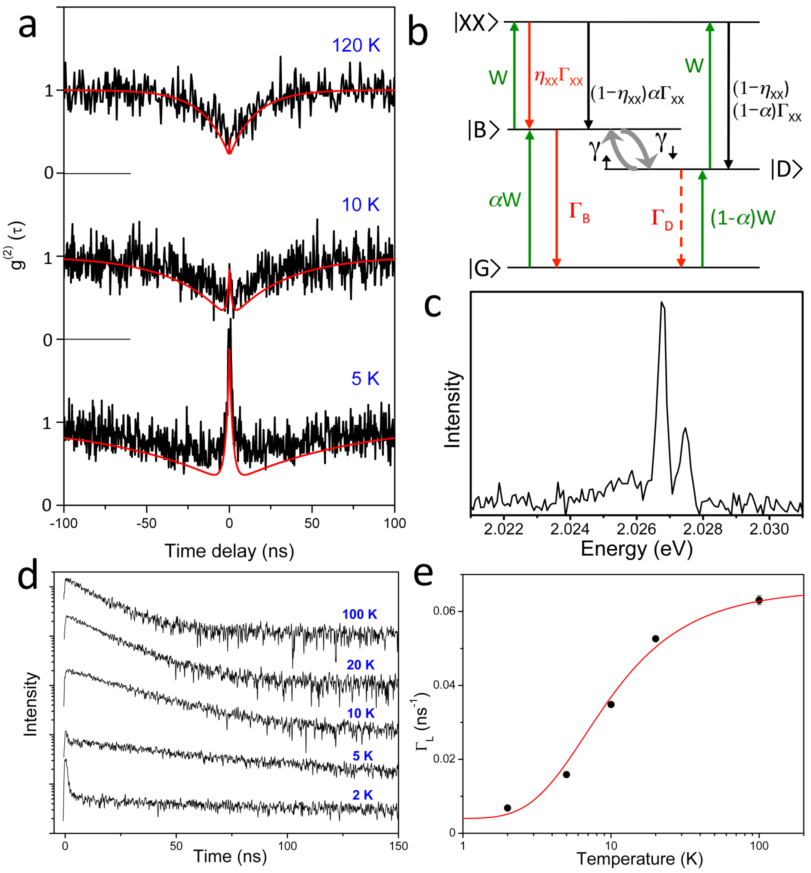

Supplementary Figure 13: Photon statistics of single CdSe NCs as a function of temperature.

a, Normalized coincidence histograms of a CdSe NC at various temperatures. The blue curves are numerical simulations of the PL autocorrelation function on the basis of a four-level model (**b**) comprising the zero-exciton ground state $|\mathrm{G}\rangle$, two excitonic states $|\mathrm{D}\rangle$ and $|\mathrm{B}\rangle$ representing the ground long-lived (so-called "dark") exciton and the lowest-energy bright exciton, with purely radiative recombination [6,7] rates Γ_D and Γ_B, respectively, and a biexciton state $|\mathrm{XX}\rangle$ which recombines into the bright exciton [8] at a rate Γ_XX. The bright and dark states are thermally mixed with a one-phonon process [9,10], using transition rates between sublevels $\gamma_\uparrow = \gamma_0^{1\varphi} N_\mathrm{B}$ and $\gamma_\downarrow = \gamma_0^{1\varphi}(N_\mathrm{B}+1)$, where $N_\mathrm{B} = 1/[\exp(\Delta_\mathrm{BD}/k_\mathrm{B}T) - 1]$ is the Bose-Einstein phonon number at the temperature T. The bright-dark splitting Δ_BD = 0.75 meV is measured from the PL spectrum of this NC (**c**). We consider equal pumping rates to $|\mathrm{D}\rangle$ and $|\mathrm{B}\rangle$ $(\alpha = 0.5)$ [9], using $W = 0.03$ ns^{-1}. **d**, Evolution of the PL decay of this NC with temperature. Adjusting the temperature dependence of its long-component decay rate Γ_L (**e**) with the expression (red curve)

$$\Gamma_\mathrm{L} = \frac{1}{2}\left\{\Gamma_\mathrm{B} + \Gamma_\mathrm{D} + \gamma_\downarrow + \gamma_\uparrow - \sqrt{(\Gamma_\mathrm{B} - \Gamma_\mathrm{D} + \gamma_\downarrow - \gamma_\uparrow)^2 + 4\gamma_\downarrow\gamma_\uparrow}\right\}$$

allows a robust derivation of its relaxation parameters $\gamma_0^{1\varphi} = 0.2$ ns^{-1}, $\Gamma_\mathrm{B} = 0.13$ ns^{-1}, $\Gamma_\mathrm{D} = 0.003$ ns^{-1}. We take a biexciton quantum yield $\eta_{XX} = 0.3$. It can be estimated from the study of the PL spectrum as a function of the excitation intensity [11] and the acquisition of coincidence histograms under pulsed excitation [11,12].

Supplementary Note 1: Estimation of the exchange interaction in cuboid-shaped perovskite NCs

Modeling the band-edge exciton fine structure should take into account the NC shape anisotropy and the possible low-symmetry phase of the underlying crystal structure. Both contributions add up and may compensate each other, leading to subtle effects on the bright triplet splittings[13,14]. Yet, the low-temperature crystallographic structure of $CsPbI_3$ NCs is unknown. Moreover, crystallographic data for bulk $CsPbI_3$ are not available at these temperatures. Even when crystallographic structures are determined for bulk materials, they are not necessarily the same for NCs. In this work, the only available information on the NCs is their dispersion in morphologies from 2D-TEM images and their room-temperature orthorhombic crystal structure, which likely remains at cryogenic temperatures. Assuming an orthorhombic crystal structure, finding a bright singlet or doublet in the PL spectrum of a $CsPbI_3$ NC requires a specific shape anisotropy that compensates the crystal field effect. Since 35% of the NCs present such PL spectra, there is little chance that this splitting compensation occurs for such a large faction of NCs. This suggests a modest contribution of the crystal structure to the fine structure splitting. We have thus assumed a cubic crystal phase in our calculations of the long-range exchange interaction in cuboid shaped NCs in the weak quantum confinement regime. For this, we have developed a variational approach based on an effective mass model for the monoelectronic states. The additional contribution of short-range exchange interaction[14], as well as the effect of dielectric confinement, are taken into account in the computed fine structure splittings.

1) Bound electron-hole pair in a cuboid-shaped NC

We previously assumed a spherical shape for the NCs, introducing a size correspondence between spherical and cubic NCs ($5.02R^3 = L^3$ where R and L are respectively the radius of the sphere and the lateral size of the cube) that gives a good match in the long range interaction and in the ground state confinement energy for strongly confined NCs [2]. Trial functions for the two-particle exciton wavefunction [15] allow interpolating between the regime of strong confinement ($R/a_\mathrm{B} \ll 1$, where a_B is the Bohr radius of the exciton) and the regime of a bulk-like Wannier exciton ($R/a_\mathrm{B} \gg 1$) . The chosen trial function takes the form $\Psi(\mathbf{r}_e, \mathbf{r}_h, r_{eh}) = N f(\mathbf{r}_e) f(\mathbf{r}_h) e^{-r_{eh}/\rho}$, where $\mathbf{r}_e$ and $\mathbf{r}_h$ are the position vectors of the electron and hole respectively, $r_{eh} = |\mathbf{r}_e - \mathbf{r}_h|$, ρ is the variational parameter that indicates the degree of spatial correlation between the electron and the hole, and N is a normalization factor. For a sphere, f is a radial function developed over spherical Bessel functions. Here, we choose for a cuboid NC $f(\mathbf{r}) = \left(\frac{8}{L_\mathrm{x} L_\mathrm{y} L_\mathrm{z}}\right)^{1/2} cos\left(\frac{\pi x}{L_\mathrm{x}}\right) cos\left(\frac{\pi y}{L_\mathrm{y}}\right) cos\left(\frac{\pi z}{L_\mathrm{z}}\right)$, where $L_\mathrm{x}, L_\mathrm{y}, L_\mathrm{z}$ are the three dimensions of the cuboid.

In the strong confinement regime, the variational parameter ρ tends towards $2a_\mathrm{B}$ [15,16] and the total energy is given by

$$E_\mathrm{tot} \to \frac{\hbar^2}{2\mu}\left(\frac{\pi^2}{L_\mathrm{x}^2} + \frac{\pi^2}{L_\mathrm{y}^2} + \frac{\pi^2}{L_\mathrm{z}^2}\right). \qquad (1)$$

In the weak confinement regime, where $L/a_\mathrm{B} \gg 1$, L being the average lateral dimension $L = V^{1/3} = \left(L_\mathrm{x} L_\mathrm{y} L_\mathrm{z}\right)^{1/3}$, the variational parameter ρ tends towards a_B (Ref. [15]) and the total energy towards the binding energy of the electron and the hole in the bulk material. The calculated energy of the ground state of the correlated electron-hole pair in Rydberg units is represented in Supplementary Fig.14 for a cube ($L_\mathrm{x} = L_\mathrm{y} = L_\mathrm{z} = L$) as a function of $\log(L/a_\mathrm{B})$.

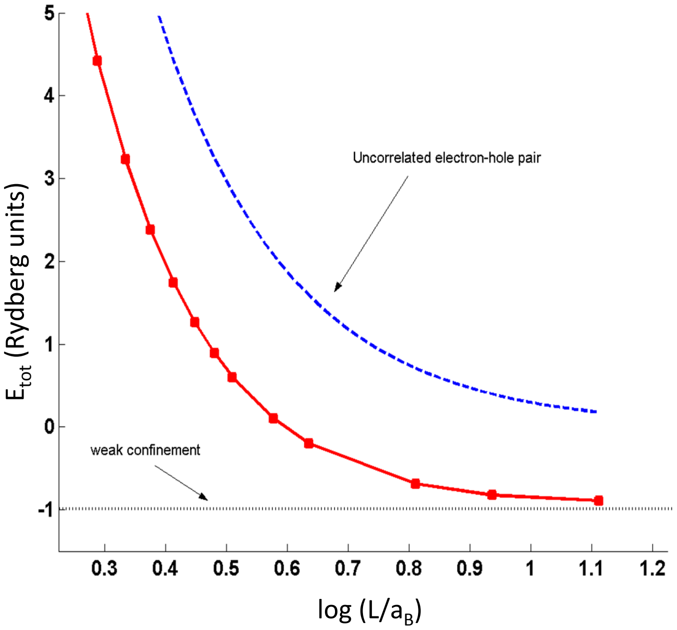

Supplementary Figure 14: Energy of the ground correlated electron-hole pair as a function of the NC size.

2) Long-range exchange interaction in cuboid NCs

The long-range part of the exchange interaction, which is very sensitive to quantum confinement, is given in the reciprocal space (wave vector **q**) by:

$$H^{\mathrm{LR}}_{m'n',mn} = \frac{1}{8\pi^3}\frac{e^2}{\varepsilon q^2}\exp\{i\mathbf{q}.(\mathbf{r}_e - \mathbf{r}_e{}')\}\delta(\mathbf{r}_e - \mathbf{r}_h)\delta(\mathbf{r}_e{}' - \mathbf{r}_h{}')\left(\sum_\alpha \sum_\beta Q^{\alpha\beta}_{m'\hat{K}n,m\hat{K}n'} q_\alpha q_\beta\right) \quad (2),$$

where $mn, m'n'$ are two pairs of conduction and valence band electronic states, $\hat{K}$ is the time reversal symmetry operator to use in the hole convention, Q a matrix containing the products of momentum operators [16,17], and ε the dielectric constant. This expression is used in the general case of anisotropic cuboids. The convergence of the **q**-space integration is checked against the results obtained from the simplified expression in the position space, valid for a spherical or cubic NC:

$$H^{\mathrm{LR}}_{m'n',mn}(\mathbf{r}'_e, \mathbf{r}'_h, \mathbf{r}_e, \mathbf{r}_h) = \frac{e^2}{3\varepsilon}\delta(\mathbf{r}_e - \mathbf{r}_e{}')\delta(\mathbf{r}_e - \mathbf{r}_h)\delta(\mathbf{r}_e{}' - \mathbf{r}_h{}')\left(\sum_\alpha Q^{\alpha\alpha}_{m'\hat{K}n,m\hat{K}n'}\right) \quad (3).$$

This expression includes the contracted form of the Q matrix, which is representative of the symmetries of the electronic Bloch functions involved in the formation of the excitonic pair states.

The exciton fine structure is directly related to the difference $\hbar\omega_{\mathrm{LT}}$ between the energies of longitudinal and transverse excitons [18]. In the bulk case, the contribution of the long-range exchange interaction to the dark singlet-bright triplet exciton splitting is thus given by [19,20]:

$$\Delta_{\mathrm{LR,BD},bulk} = \frac{\hbar\omega_{\mathrm{LT}}}{3}, \quad \text{with } \hbar\omega_{\mathrm{LT}} = \frac{2}{3\pi\varepsilon a_B^3}\left(\frac{e\hbar p_{\mathrm{cv}}}{m_0 E_{\mathrm{g}}}\right)^2 \quad (4)$$

where $p_{\mathrm{cv}} = \langle \mathrm{S}|\widehat{p_{\mathrm{z}}}|\mathrm{Z}\rangle = \langle \mathrm{S}|\widehat{p_{\mathrm{y}}}|\mathrm{Y}\rangle = \langle \mathrm{S}|\widehat{p_{\mathrm{x}}}|\mathrm{X}\rangle$ is the Kane interband momentum matrix element.

a) <u>Evolution of the long-range exchange interaction with the NC size</u>

Bright-dark splittings $\Delta_{\mathrm{LR,BD}}$ of cubic NCs are computed for different NC sizes using the trial functions defined in the first section. The calculated values are presented as a function of the emission energy (See Supplementary Fig. 15). The material parameters to evaluate the long-

range exchange interaction for $CsPbI_3$ are extracted from Ref. [21], leading to the exciton binding energy 15 meV, the energy band gap 1.723 eV, the exciton effective mass μ= 0.114 in electron-mass unit and the bulk Bohr exciton radius a_B = 4.6 nm. For MA- and FA-based hybrid perovskites, material parameters are obtained from Ref. [22].

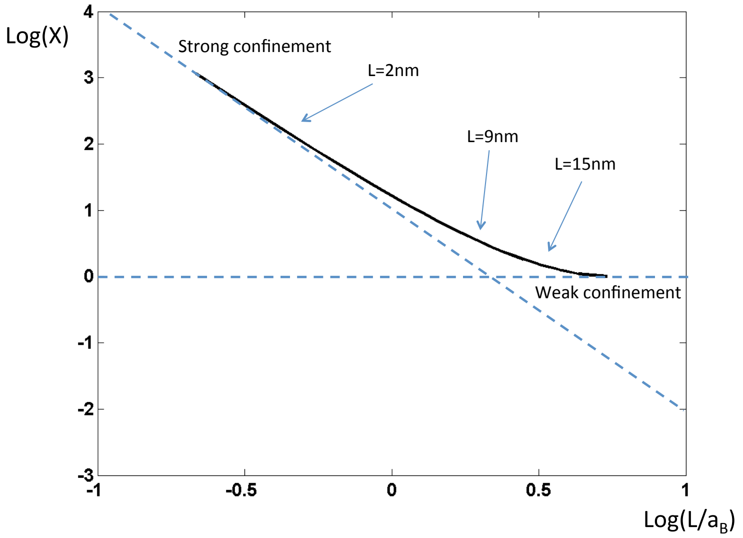

Supplementary Figure 15: Size dependence of the long-range exchange interaction.
$X = \Delta_{\mathrm{LR,BD}}/\Delta_{\mathrm{LR,BD},bulk}$. The dashed lines correspond to strong and weak confinement regimes. The values of L are given in the case of $CsPbI_3$ NCs (a_B = 4.6 nm) [21]. The mean size of these NCs (11.2 ± 1.2 nm) sets the exciton in a regime of weak confinement.

b) Short-range exchange interaction

The short-range contribution of the exchange interaction is added to the long-range contribution to compute the bright-dark splitting. In Fig. 3f, the bulk short-range exchange interaction of (MA/FA)-Pb-(I/Br)$_3$ perovskites are taken from Ref.[14]. For $CsPbI_3$ and $CsPbBr_3$ perovskites, the bulk short-range values are deduced from their hybrid organic-inorganic perovskite counterparts by applying a $1/a_B^3$ scale law[14]. For instance, the bulk SR value is 0.139 meV for $CsPbI_3$, starting from the bulk short-range value of 0.081 meV given for $MAPbI_3$.[14] It adds to the LR contribution of 0.613 meV, leading to a total bulk exchange interaction of 0.752 meV. Taking into account the SR contribution in the bright-dark splitting of $CsPbI_3$ NCs results in multiplying the splittings calculated with the LR contribution alone by a factor of 1.227, which is done in Fig. 3.

c) Dielectric confinement effect

The dielectric confinement likely plays a role on the exchange interaction. Using a dielectric constant $\varepsilon_{r,\mathrm{NC}}$ = 6.05 for $CsPbI_3$ [23] and $\varepsilon_{r,\mathrm{ext}}$ for the NC external medium, a correction factor $1 + \frac{12(\varepsilon_{r,\mathrm{NC}}/\varepsilon_{r,\mathrm{ext}}-1)}{\pi^2(\varepsilon_{r,\mathrm{NC}}/\varepsilon_{r,\mathrm{ext}}+2)}$, valid for the weak confinement regime (Ref. [24]), is applied to the dark-bright splitting calculated in section a) (see Fig. 3e). Since the studied NCs are embedded in a polymer layer with a thickness of few tens of nanometers located between vacuum and a sapphire coverslip, the dielectric constant of the external medium is difficult to determine and may differ from one NC to another. In Fig. 3b, we consider the two extreme dielectric-confinement correction factors 1.763 and 1.289 respectively associated to the external dielectric constants $\varepsilon_{r,\mathrm{ext}}$ =1 (for NCs located at the vacuum-polymer interface) and $\varepsilon_{r,\mathrm{ext}} = 3.126$ (for NCs deeply embedded in the polymer layer, close to the sapphire coverslip).

Supplementary Note 2: Four-level model for the calculation of the PL intensity autocorrelation function

The simulated PL intensity autocorrelation function is calculated from the solutions of rate equations in a simple four-level model including the zero-exciton ground level, thermally mixed bright and dark exciton sublevels and a biexciton level, as shown in Supplementary Fig. 14. The thermal mixing model involves a two-LO phonon process in perovskites NCs and a single acoustic phonon process in CdSe NCs.

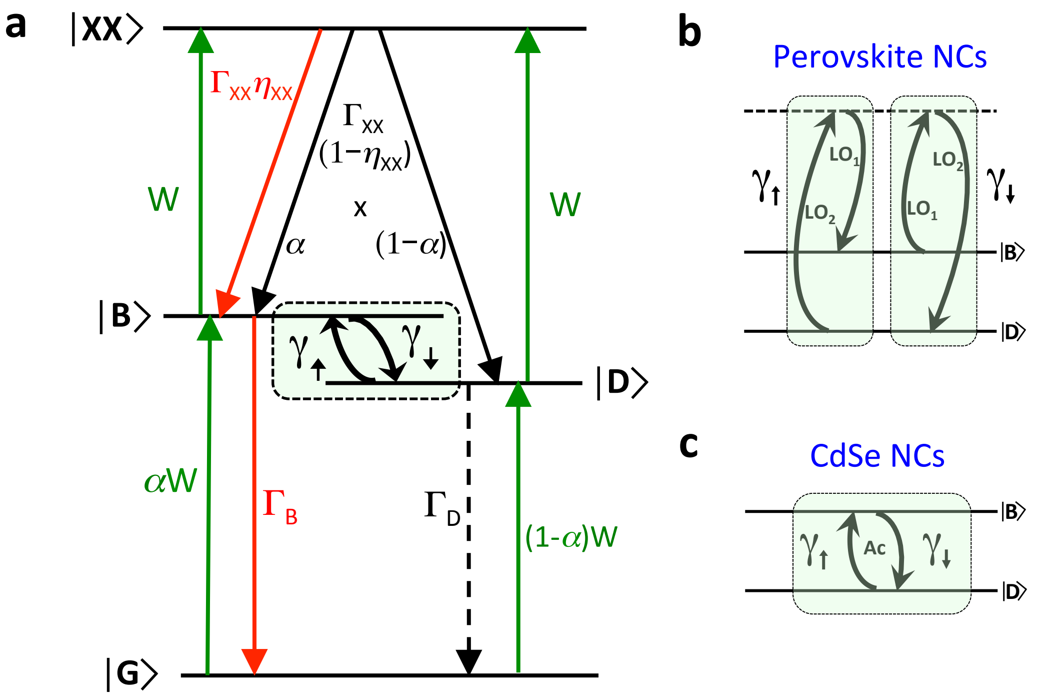

Supplementary Figure 16: One-phonon and two-phonon thermal mixing models of bright and dark exciton states.

a, The four-level system comprises the zero-exciton ground level $|G\rangle$, thermally mixed dark and bright exciton sublevels $|D\rangle, |B\rangle$ with relaxation rates Γ_D and Γ_B, respectively, and a biexciton level $|XX\rangle$ with a relaxation rate $\Gamma_{XX} \sim 4\Gamma_B/\eta_{XX}$.[25] W is the pumping rate and α denotes the relative degeneracy of bright and dark exciton states. **b**, In the case of perovskite NCs, with α=3/4, the transition rates $\gamma_\downarrow$ and $\gamma_\uparrow$ between dark and bright states involve the absorption and emission of phonons (for instance two optical phonons named LO_1, LO_2) whose energy difference matches the bright-dark splitting Δ_{BD}. The transition rates are $\gamma_\uparrow = \gamma_0 N_2(N_1 + 1)$ and $\gamma_\downarrow = \gamma_0 N_1(N_2 + 1)$, where $N_i = 1/[\exp(E_i/k_B T) - 1]$ with i = 1, 2 are the Bose-Einstein phonon numbers at temperature T, and γ_0 is a characteristic two-phonon mixing rate. **c**, In the case of CdSe NCs, with α=1/2, thermal mixing involves the emission and absorption of acoustic phonons from a mode whose energy matches the bright-dark splitting. The transition rates are $\gamma_\uparrow = \gamma_0^{1\varphi} N_B$ and $\gamma_\downarrow = \gamma_0^{1\varphi}(N_B + 1)$, where $N_B = 1/[\exp(\Delta_{BD}/k_B T) - 1]$.

The simulated autocorrelation function is deduced from the solutions of rate equations:

$$\begin{cases} \dot{p_{\mathrm{G}}}(t) = -Wp_{\mathrm{G}} + \Gamma_{\mathrm{B}}p_{\mathrm{B}} + \Gamma_{\mathrm{D}}p_{\mathrm{D}} \\ \dot{p_{\mathrm{B}}}(t) = \alpha Wp_{\mathrm{G}} - (\gamma_{\downarrow} + \Gamma_{\mathrm{B}} + W)p_{\mathrm{B}} + \gamma_{\uparrow}p_{\mathrm{D}} + \Gamma_{\mathrm{XX}}[\eta_{\mathrm{XX}} + (1-\eta_{\mathrm{XX}})\alpha]p_{\mathrm{XX}} \\ \dot{p_{\mathrm{D}}}(t) = (1-\alpha)Wp_{\mathrm{G}} + \gamma_{\downarrow}p_{\mathrm{B}} - (\gamma_{\uparrow} + \Gamma_{\mathrm{D}} + W)p_{\mathrm{D}} + \Gamma_{\mathrm{XX}}(1-\eta_{\mathrm{XX}})(1-\alpha)p_{\mathrm{XX}} \\ \dot{p_{\mathrm{XX}}}(t) = W(p_{\mathrm{B}} + p_{\mathrm{D}}) - \Gamma_{\mathrm{XX}}p_{\mathrm{XX}} \end{cases}$$

where $p_{\mathrm{G}}(t)$, $p_{\mathrm{D}}(t)$, $p_{\mathrm{B}}(t)$ and $p_{\mathrm{XX}}(t)$ are the populations at time t of ground, dark, bright and biexciton states, respectively.

The calculation of the autocorrelation function takes into account all possible correlations between a photon stemming from state j at time τ and a photon stemming from state i at time $t = 0$, where $i, j = \mathrm{D}, \mathrm{B}, \mathrm{XX}$. The emission rates from these states are $\eta_{\mathrm{i}}\Gamma_i p_{\mathrm{i}}$, η_{i} being their radiative quantum yields. We take $\eta_{\mathrm{D}} = 0$ for perovskite NCs and $\eta_{\mathrm{D}} = 1$ for CdSe NCs, $\eta_{\mathrm{B}} = 1$, and η_{XX} is an adjustable parameter. The disparity in biexciton quantum yields among the NCs[11] may arise from their various morphologies and surface states, which affect the nonradiative Auger recombination processes. The normalized autocorrelation function $g^{(2)}(\tau)$ at the time delay τ is given by:

$$g^{(2)}(\tau) = \Big\{[\Gamma_{\mathrm{B}}p_{\mathrm{B}}(\infty) + \eta_{\mathrm{D}}\Gamma_{\mathrm{D}}p_{\mathrm{D}}(\infty)][\Gamma_{\mathrm{B}}p_{\mathrm{B}}^{\mathrm{G}(0)}(\tau) + \eta_{\mathrm{D}}\Gamma_{\mathrm{D}}p_{\mathrm{D}}^{\mathrm{G}(0)}(\tau) + \eta_{\mathrm{XX}}\Gamma_{\mathrm{XX}}p_{\mathrm{XX}}^{\mathrm{G}(0)}(\tau)]$$
$$+\eta_{\mathrm{XX}}\Gamma_{\mathrm{XX}}p_{\mathrm{XX}}(\infty)[\Gamma_{\mathrm{B}}p_{B}^{\mathrm{B}(0)}(\tau) + \eta_{\mathrm{D}}\Gamma_{\mathrm{D}}p_{\mathrm{D}}^{\mathrm{B}(0)}(\tau) + \eta_{\mathrm{XX}}\Gamma_{\mathrm{XX}}p_{\mathrm{XX}}^{\mathrm{B}(0)}(\tau)]\Big\}$$
$$/\{\Gamma_{\mathrm{B}}p_{\mathrm{B}}(\infty) + \eta_{\mathrm{D}}\Gamma_{\mathrm{D}}p_{\mathrm{D}}(\infty) + \eta_{\mathrm{XX}}\Gamma_{\mathrm{XX}}p_{\mathrm{XX}}(\infty)\}^2$$

where $p_i(\infty)$ is the stationary population of state i and $p_i^{j(0)}(\tau)$ the population of state i at time τ, knowing that the system is in the state j at zero time.

Supplementary References

1. Fu, M. *et al.* Neutral and Charged Exciton Fine Structure in Single Lead Halide Perovskite Nanocrystals Revealed by Magneto-Optical Spectroscopy. *Nano Lett.* **17,** 2895–2901 (2017).
2. Tamarat, P. *et al.* The ground exciton state of formamidinium lead bromide perovskite nanocrystals is a singlet dark state. *Nat Mater* **18,** 717–724 (2019).
3. Pérez-Osorio, M. A. *et al.* Vibrational Properties of the Organic–Inorganic Halide Perovskite $CH_3NH_3PbI_3$ from Theory and Experiment: Factor Group Analysis, First-Principles Calculations, and Low-Temperature Infrared Spectra. *J. Phys. Chem. C* **119,** 25703–25718 (2015).
4. Ferreira, A. C. *et al.* Direct evidence of weakly dispersed and strongly anharmonic optical phonons in hybrid perovskites. *Communications Physics* **3,** 48 (2020).
5. Fu, M. *et al.* Unraveling exciton–phonon coupling in individual $FAPbI_3$ nanocrystals emitting near-infrared single photons. *Nature Communications* **9,** 3318 (2018).
6. Biadala, L., Louyer, Y., Tamarat, P. & Lounis, B. Direct Observation of the Two Lowest Exciton Zero-Phonon Lines in Single CdSe/ZnS Nanocrystals. *Physical Review Letters* **103,** 037404 (2009).
7. Fernée, M. J., Tamarat, P. & Lounis, B. Cryogenic Single-Nanocrystal Spectroscopy: Reading the Spectral Fingerprint of Individual CdSe Quantum Dots. *J. Phys. Chem. Lett.* **4,** 609–618 (2013).
8. Rodina, A. V. & Efros, A. L. Band-edge biexciton in nanocrystals of semiconductors with a degenerate valence band. *Phys. Rev. B* **82,** 772 (2010).
9. Labeau, O., Tamarat, P. & Lounis, B. Temperature Dependence of the Luminescence Lifetime of Single CdSe/ZnS Quantum Dots. *Physical Review Letters* **90,** 257404 (2003).
10. Louyer, Y., Biadala, L., Tamarat, P. & Lounis, B. Spectroscopy of neutral and charged exciton states in single CdSe/ZnS nanocrystals. *Appl. Phys. Lett.* **96,** 203111 (2010).
11. Louyer, Y. *et al.* Efficient Biexciton Emission in Elongated CdSe/ZnS Nanocrystals. *Nano Lett.* **11,** 4370–4375 (2011).
12. Nair, G., Zhao, J. & Bawendi, M. G. Biexciton Quantum Yield of Single Semiconductor Nanocrystals from Photon Statistics. *Nano Lett.* **11,** 1136–1140 (2011).
13. Ben Aich R *et al.* Bright-Exciton Splittings in Inorganic Cesium Lead Halide Perovskite Nanocrystals. *Phys. Rev. Applied* **11,** 034042 (2019).
14. Ben Aich R, Ben Radhia S, Boujdaria, K., Chamarro, M. & Testelin, C. Multiband k·p Model for Tetragonal Crystals: Application to Hybrid Halide Perovskite Nanocrystals. *J. Phys. Chem. Lett.* **11,** 808–817 (2020).
15. Kayanuma, Y. Wannier Exciton in Microcrystals. *Solid State Commun.* **59,** 405–408 (1986).
16. Goupalov, S. V. & Ivchenko, L. E. Electron-hole long-range exchange interaction in semiconductor quantum dots. *Journal of Crystal Growth* **184/185,** 393–397 (1998).
17. Pikus, G. E. & Bir, G. L. Exchange interaction in excitons in semiconductors. *Sov Phys JETP* **33,** 108–114 (1971).
18. Pikus, G. E. & Bir, G. L. Exchange Interaction in Bound Excitons in Semiconductors. *Soviet Physics JETP* **35,** 174–178 (1972).
19. Tong, H. & Wu, M. W. Theory of excitons in cubic III-V semiconductor GaAs, InAs and GaN quantum dots: Fine structure and spin relaxation. *Phys. Rev. B* **83,** 235323 (2011).
20. Nestoklon, M. O. *et al.* Optical orientation and alignment of excitons in ensembles of inorganic perovskite nanocrystals. *Phys. Rev. B* **97,** 235304 (2018).
21. Yang, Z. *et al.* Impact of the Halide Cage on the Electronic Properties of Fully Inorganic Cesium Lead Halide Perovskites. *ACS Energy Lett.* **2,** 1621–1627 (2017).
22. Galkowski, K. *et al.* Determination of the exciton binding energy and effective masses for

methylammonium and formamidinium lead tri-halide perovskite semiconductors. *Energy & Environmental Science* **9,** 962–970 (2016).
23. Singh, R. K. *et al.* Investigation of optical and dielectric properties of $CsPbI_3$ inorganic lead iodide perovskite thin film. *Journal of the Taiwan Institute of Chemical Engineers* **96,** 538 (2018).
24. Sercel, P. C. *et al.* Exciton Fine Structure in Perovskite Nanocrystals. *Nano Lett.* **19,** 4068–4077 (2019).
25. Park, Y.-S., Bae, W. K., Pietryga, J. M. & Klimov, V. I. Auger Recombination of Biexcitons and Negative and Positive Trions in Individual Quantum Dots. *ACS Nano* **8,** 7288 (2014).
